# Body mass index and fasting insulin predict survival and EGFR–TKI benefit in Stage IV lung adenocarcinoma

**DOI:** 10.3389/fonc.2026.1742177

**Published:** 2026-06-25

**Authors:** Huiru Guo, Lingshuang Liu, Jan P. A. Baak

**Affiliations:** 1 Department VI of Medical Oncology, Longhua University Hospital, Shanghai, China; 2 Department of Molecular Digital Pathology, Stavanger University Hospital, Stavanger, Norway

**Keywords:** body mass index, cancer metabolism, EGFR tyrosine kinase inhibitor, fasting insulin (FINS), lung adenocarcinoma, non-small cell lung cancer, overall survival (OS time)

## Abstract

**Background:**

Obesity, hyperinsulinemia, and type 2 diabetes mellitus (T2DM) are considered adverse metabolic conditions in cancer, although exceptions like the “obesity paradox” have been reported. Their prognostic significance in advanced lung adenocarcinoma (LUAD), particularly in patients receiving tyrosine kinase inhibitor (TKI) therapy, remains unclear.

**Methods:**

We prospectively analyzed 133 consecutive patients with newly diagnosed stage IV LUAD and ECOG performance status 0–1 enrolled between 2020 and 2024. All had ≥3 months of follow-up and T2DM status. BMI was available in 104 patients, glucose and fasting insulin in 46 patients. Primary endpoint was overall survival (OS), assessed using Kaplan–Meier and multivariable Cox regression. Propensity score and interaction analyses evaluated TKI, immune checkpoint inhibitor (ICI), and anti-VEGF therapies.

**Results:**

High BMI, hyperglycemia, hyperinsulinemia, and T2DM were not associated with worse outcomes. In contrast, low BMI (<22.1 kg/m²; *P* = 0.007, HR 2.56, 95%CI 1.29 - 5.08) and low fasting insulin (≤34 pmol/L; *P* < 0.001, HR 10.02, 95%CI 2.73–36.8) were independently associated with shorter OS, while EGFR–TKI therapy was associated with improved survival (*P* = 0.02, HR 0.52). Patients with combined BMI <22.1 kg/m² and insulin <34 pmol/L also derived survival benefit from EGFR–TKI therapy; however, absence of EGFR–TKI in this subgroup was associated with very poor 3-year OS (35%). ICI and anti-VEGF outcomes were not significantly affected.

**Conclusions:**

Low BMI and low fasting insulin identify a metabolically depleted subgroup with poor prognosis in stage IV LUAD, with worse outcomes observed in patients not receiving EGFR–TKI therapy. These parameters may improve risk stratification and support early nutritional and exercise-based interventions.

## Introduction

Non–small cell lung cancer (NSCLC) remains the leading cause of cancer-related mortality worldwide, accounting for more than 1.6 million deaths annually. NSCLC represents the majority of lung cancer cases, with many patients presenting with stage IV disease, most commonly lung adenocarcinoma (LUAD), the predominant histological subtype (1, 2). Despite advances in systemic therapy, long-term survival remains poor (3–5).

Current treatment strategies are guided by tumor-derived biomarkers, including EGFR and ALK alterations, PD-L1 expression, and tumor mutational burden (TMB). Although these markers improve patient stratification, they do not fully explain variability in treatment response and survival.

Metabolic reprogramming is a hallmark of cancer. The Warburg effect—tumor reliance on aerobic glycolysis—underlies 18F-FDG positron emission tomography (PET), a standard imaging modality in NSCLC staging (6, 7). In addition to tumor-intrinsic metabolism, systemic metabolic factors such as glucose homeostasis, insulin signaling, and energy balance may influence cancer progression and treatment response (8–13).

Clinical data support the relevance of host metabolic status. Perioperative metabolic optimization strategies, including preoperative carbohydrate loading and enhanced recovery after surgery (ERAS) protocols, improve metabolic homeostasis and clinical outcomes in colorectal surgery (14–16). However, such interventions may also influence tumor biology and proliferation in certain operable cancers (17, 18), suggesting that systemic energy balance may affect cancer behavior and treatment tolerance.

Experimental studies indicate that dysregulated glucose metabolism promotes LUAD progression (19, 20). Epidemiological evidence further supports a role for systemic metabolic factors. Higher BMI has been associated with improved survival in advanced NSCLC, a phenomenon often referred to as the “obesity paradox” (21). In contrast, type 2 diabetes mellitus (T2DM) and poor glycemic control have been associated with worse outcomes, although findings remain inconsistent (22). Elevated fasting glucose has been linked to shorter survival (8, 23), whereas metformin use has been associated with improved outcomes in patients with diabetes and advanced disease (24). BMI may also influence response to immune checkpoint inhibitors, and combined EGFR–TKI and metformin therapy has shown potential survival benefits in LUAD (25–27).

Mechanistically, BMI, glycemic status, insulin signaling, and metformin exposure may influence tumor progression and treatment response through insulin/IGF-1 pathways, altered glucose availability, and modulation of the tumor microenvironment.

Despite these observations, the prognostic impact of metabolic factors in stage IV LUAD remains unclear. Prior studies have included heterogeneous populations with mixed histology or disease stages, limiting interpretability. In addition, while metabolic excess has been widely studied, the potential impact of metabolic depletion has received less attention.

We hypothesized that both metabolic excess and metabolic depletion differentially influence survival and treatment response in advanced LUAD.

We therefore conducted a prospective analysis of a consecutive, clinically homogeneous cohort of patients with stage IV LUAD, ECOG performance status 0–1, and ≥3 months of follow-up. We evaluated the prognostic and treatment-modifying effects of BMI, glycemic parameters, and insulin-related factors on survival and response to EGFR–TKI therapy, and to determine whether metabolic depletion identifies a subgroup of patients with particularly poor survival, especially in relation to treatment selection and outcomes in patients not receiving targeted therapy.

## Methods

### Study design and patients

This was a prospective, observational, non-interventional cohort study conducted at Longhua University Hospital (LUH), Shanghai, China, enrolling patients with stage IV LUAD and ECOG performance status 0–1. The study protocol was approved by the LUH Institutional Review Board (IRB 2022LCSY024) and conducted in accordance with international ethical guidelines (28, 29). Written informed consent was obtained from all participants.

Between January 1, 2020, and December 31, 2024, consecutive patients with pathologically confirmed stage IV NSCLC were screened. Eligible patients had histologically confirmed LUAD, ECOG performance status 0–1, available baseline blood samples at diagnosis, and at least 3 months of follow-up. Patients with ECOG PS 2–4, non-adenocarcinoma histology, incomplete baseline data, or follow-up shorter than 3 months were excluded. Of 385 screened patients, 133 consecutive stage IV LUAD cases met eligibility criteria (see **Supplementary Figure 1**; STROBE checklist).

### Data collection and follow-up

Baseline demographics, TNM stage (IVA vs IVB) (30), baseline treatment information, and molecular biomarker data (EGFR and other oncogenic drivers) were extracted from electronic medical records. Follow-up was performed monthly through clinic visits or structured telephone follow-up until March 31, 2025.

### Treatments

Patients received standard-of-care therapies, including platinum-based chemotherapy (31), EGFR–TKIs, immune checkpoint inhibitors (ICIs), and bevacizumab, administered alone or in combination. The most commonly used EGFR–TKIs were gefitinib erlotinib, followed by Osimertinib. TKI agents other than EGFR-TKI (n=24) consisted of 5 different types, 3 of which consisted of very few patients (**Supplementary Table 1**) Therefore, treatment was analyzed as a binary variable (EGFR–TKI use: yes/no), where EGFR-TKI = no (n=56) consisted of 2 subgroups: No TKI at all (n=32) and No EGFR-TKI (n=24). Palliative radiotherapy was delivered as clinically indicated (32).

Treatment variables were not included in baseline prognostic analyses, as therapies were initiated after biomarker assessment. Sensitivity analyses indicated that treatment heterogeneity did not materially affect the main findings.

### Baseline variables

Baseline variables included age, sex, TNM stage (IVA vs IVB), BMI (weight in kilograms divided by height in meters with 2 decimals), glucose, insulin, ferritin (as a marker of systemic inflammation), T2DM status, and EGFR mutation status. ECOG performance status was not included in further analyses because all patients met the inclusion criterion (ECOG 0–1).

### Blood samples

Baseline serum samples were collected at the first LUH visit, prior to initiation of systemic therapy. Glucose, ferritin and fasting insulin were measured in the LUH clinical laboratory using standardized immunoassays; details of assay platforms and technical aspects are provided in the **Supplementary Table 2**.

### Statistical analysis

Analyses were performed using SPSS version 29.0 (IBM Corp., Armonk, NY, USA) and MedCalc version 23.03.7 (MedCalc Software, Ostend, Belgium). The primary endpoint was overall survival (OS), defined as the time from diagnosis to death from any cause. All analyses adhered to STROBE reporting guidelines (see **Supplementary Figure 1**).

Survival curves were estimated using the Kaplan–Meier method and compared using log-rank tests. Cox proportional hazards regression models were used to evaluate prognostic associations, with interaction terms included to assess potential effect modification by treatment. The proportional hazards assumption was formally tested for all variables.

BMI was categorized according to established World Health Organization (WHO) criteria. For the general population, BMI categories were defined as: underweight (<18.5 kg/m²), normal weight (18.5–24.9 kg/m²), overweight (25.0–29.9 kg/m²), and obesity (≥30.0 kg/m²). For Asian populations, lower WHO-recommended thresholds were applied: underweight (<18.5 kg/m²), normal weight (18.5–22.9 kg/m²), overweight (23.0–27.4 kg/m²), and obesity (≥27.5 kg/m²). Both classification systems were analyzed.

The normal glucose, ferritin and fasting insulin range were 3.9-6.1 mmol/L, for ferritin in men: 30 – 400 µg/L and in women 13 – 150 µg/L, and for fasting insulin 18–173 pmol/L (For details of assay methods and normal reference ranges for insulin, ferritin, and sIL-2R, see **Supplementary Table 2**).

BMI and other continuous variables (insulin, glucose, and ferritin) were analyzed as both continuous and dichotomous variables. Dichotomization thresholds were determined using receiver operating characteristic (ROC) curve analysis, with optimal cut-offs identified by the Youden index. The ROC-derived BMI cutoff (22.1 kg/m²) and for insulin 34.0 pmol/L were used as the primary threshold. To evaluate robustness and reduce potential overfitting, additional analyses were performed using median- and tertile-based categorizations.

Multivariable Cox regression models included age, sex, TNM stage, EGFR mutation status, BMI, insulin, glucose, type 2 diabetes mellitus (T2DM), and ferritin. Interaction terms between metabolic variables and treatment modalities (EGFR–TKIs, immune checkpoint inhibitors [ICIs], and anti-VEGF therapies) were included to explore effect modification.

To mitigate confounding, propensity scores were estimated from baseline covariates using 1:1 nearest-neighbor matching with a caliper of 0.1. Covariate balance was assessed using standardized mean differences (SMD), with values <0.1 indicating acceptable balance (**Supplementary Tables 3**, **4**). Sensitivity analyses confirmed consistent results before and after matching (see **Supplementary File**) (31–38).

Missing BMI and insulin data were not imputed in the primary analysis for descriptive statistics; all analyses were conducted using complete-case data. Sensitivity analyses were performed using pragmatic substitution approaches (median substitution and a conservative low-value scenario) to assess the robustness of the findings. These analyses were not intended to provide unbiased imputed estimates but to evaluate consistency in the direction and magnitude of associations under different assumptions regarding missing data.

Analyses were conducted using available data for each variable, and the number of patients included in each analysis is reported accordingly. All statistical tests were two-sided, and P < 0.05 was considered statistically significant.

## Results

### Patient characteristics

Between January 2020 and December 2024, 385 consecutive patients with stage IV non–small cell lung cancer were screened. Of these, 133 patients with histologically confirmed lung adenocarcinoma (LUAD), ECOG performance status 0–1, and at least 3 months of follow-up were included (**Supplementary Figure 1**). The median follow-up time was 24.1 months (range, 3.0–60.0 months). The median age was 67 years (range, 35–92), 62% were male, and 52 patients (39%) had died at the time of analysis.

Baseline clinical and metabolic characteristics are summarized in **Table 1**.

**Table 1:** Univariate analysis of overall survival in stage IV LUAD (n=133).

Variable	Events/Total	Median OS (months)	HR (95% CI)	P value
Age (<68 vs ≥68)	25/74 vs 27/59	52.0 vs 31.0	2.17 (1.22–3.88)	0.009
Sex (female vs male)	17/52 vs 35/81	60.0 vs 45.0	1.64 (0.93–2.87)	0.09
BMI (<22.1 vs 22.1-33.9 kg/m²)*	16/58 vs 21/46	31.0 vs NE	2.56 (1.29–5.08)	0.007
Diabetes mellitus (yes vs no)	8/18 vs 44/115	31.0 vs 49.0	1.79 (0.72–4.47)	0.21
TNM stage (IVB vs IVA)	30/73 vs 22/60	49.0 vs 60.0	1.28 (0.73–2.24)	0.38
EGFR mutation (no/other vs yes)	27/56 vs 23/71	30.0 vs 49.0	1.93 (1.07–3.46)	0.03
Ferritin (≥328 vs <328 ng/mL)	31/60 vs 21/73	31.0 vs NE	2.02 (1.15–3.54)	0.01
Glucose (>5.64 vs ≤5.64 mmol/L)**	8/13 vs 12/36	30.0 vs NE	2.55 (0.89–7.28)	0.08
Insulin (≤34 vs >34 pmol/L)***	8/11 vs 11/35	15.0 vs NE	10.02 (2.73–36.8)	<0.001
EGFR–TKI therapy (yes vs no)	23/71 vs 29/62	30.0 vs 49.0	0.52 (0.29–0.93)	0.02
Immunotherapy (yes vs no)	17/38 vs 35/95	39.0 vs 52.0	1.22 (0.66–2.24)	0.53
Antiangiogenic therapy (yes vs no)	26/48 vs 26/85	39.0 vs 60.0	1.37 (0.78–2.39)	0.28
Chemotherapy cycles (≥4 vs <4)	36/76 vs 16/57	39.0 vs 60.0	1.78 (1.02–3.11)	0.04

*BMI available in 104 patients; **Glucose in 49 patients; ***Insulin in 46 patients. NE, not estimable; HR, hazard ratio; CI, confidence interval; OS, overall survival.

### Univariable survival analysis

The range of BMI in the population was 16.5-33.9 kg/m^2^ (median: 22.6). In univariate analyses, younger age (<68 years; P = 0.009), higher BMI (≥22.1 kg/m²; P = 0.007), and EGFR-mutation positivity (P = 0.03) were significantly associated with *improved* OS. Female sex showed a non-significant trend toward improved OS (P = 0.09). Classification accuracy of BMI <22.1 vs ≥ 22.1 of the 104 patients with known BMI in the whole patients´ cohort (n=104) is shown in **Supplementary Table 5**.

Body mass index (BMI) was significantly associated with overall survival. When analyzed according to World Health Organization (WHO) BMI categories, underweight patients had significantly poorer survival compared to those with BMI ≥18.5 kg/m² (median 15 vs. 49 months, P = 0.04). In contrast, higher BMI (≥25 kg/m²) was not associated with worse outcomes and showed a non-significant trend toward improved survival. Analyses using both general and Asian-specific WHO BMI classifications yielded consistent patterns, although the number of patients in extreme BMI categories was limited. Survival estimates across categories should therefore be interpreted with caution due to small subgroup sizes.

**Figure 1:**
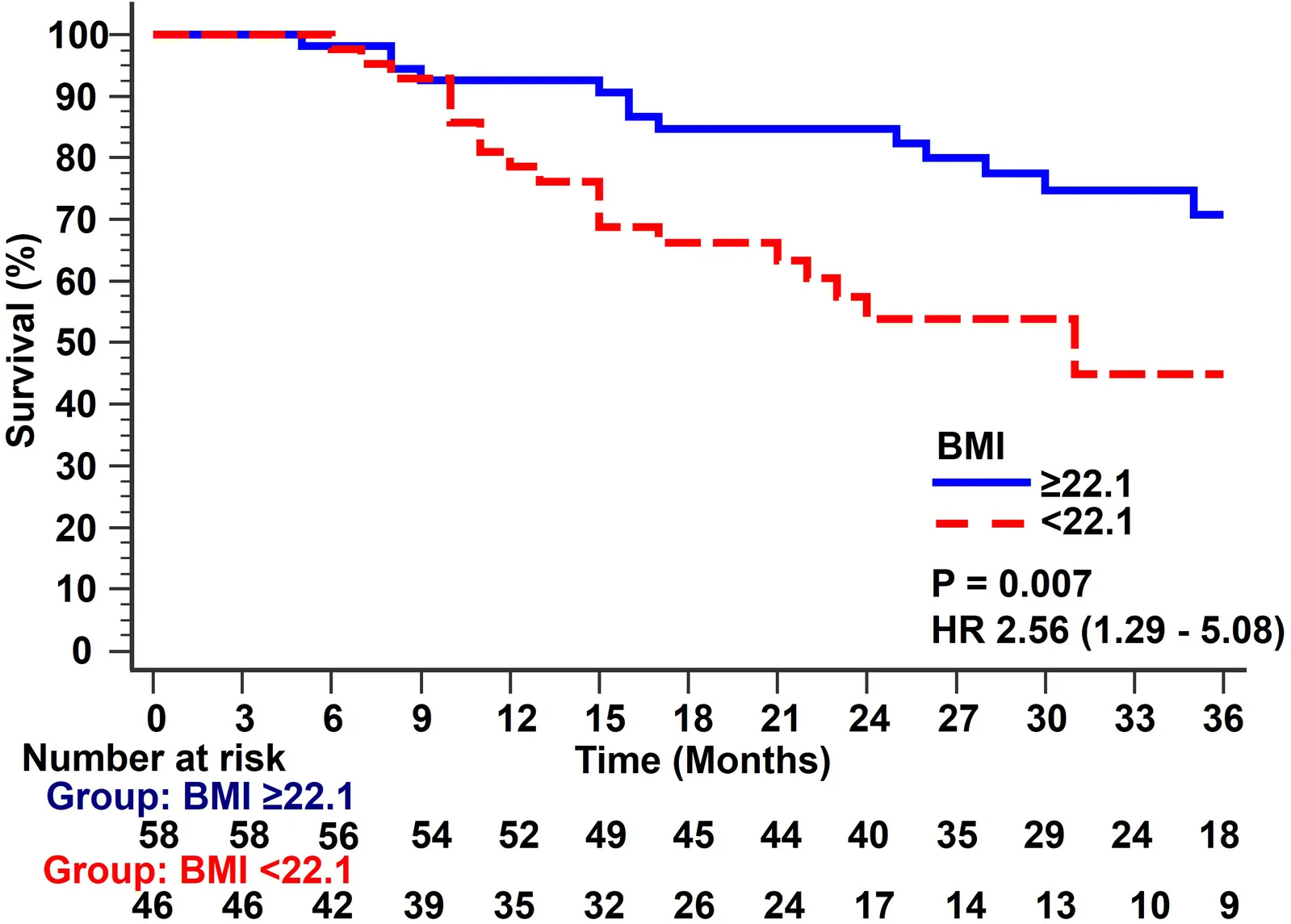
Kaplan-Meier survival curves of the patients stratified over BMI ≥22.1 versus <22.1 in all 104 patients with available BMI data.

High BMI, hyperglycemia, hyperinsulinemia, and type 2 diabetes mellitus (T2DM) were not associated with worse outcomes (**Table 1**). In contrast, BMI <22.1 kg/m² was significantly associated with shorter OS than those with BMI ≥ 22.1. Kaplan–Meier analysis showed clear separation between groups (**Figure 1**).

**Figure 2:**
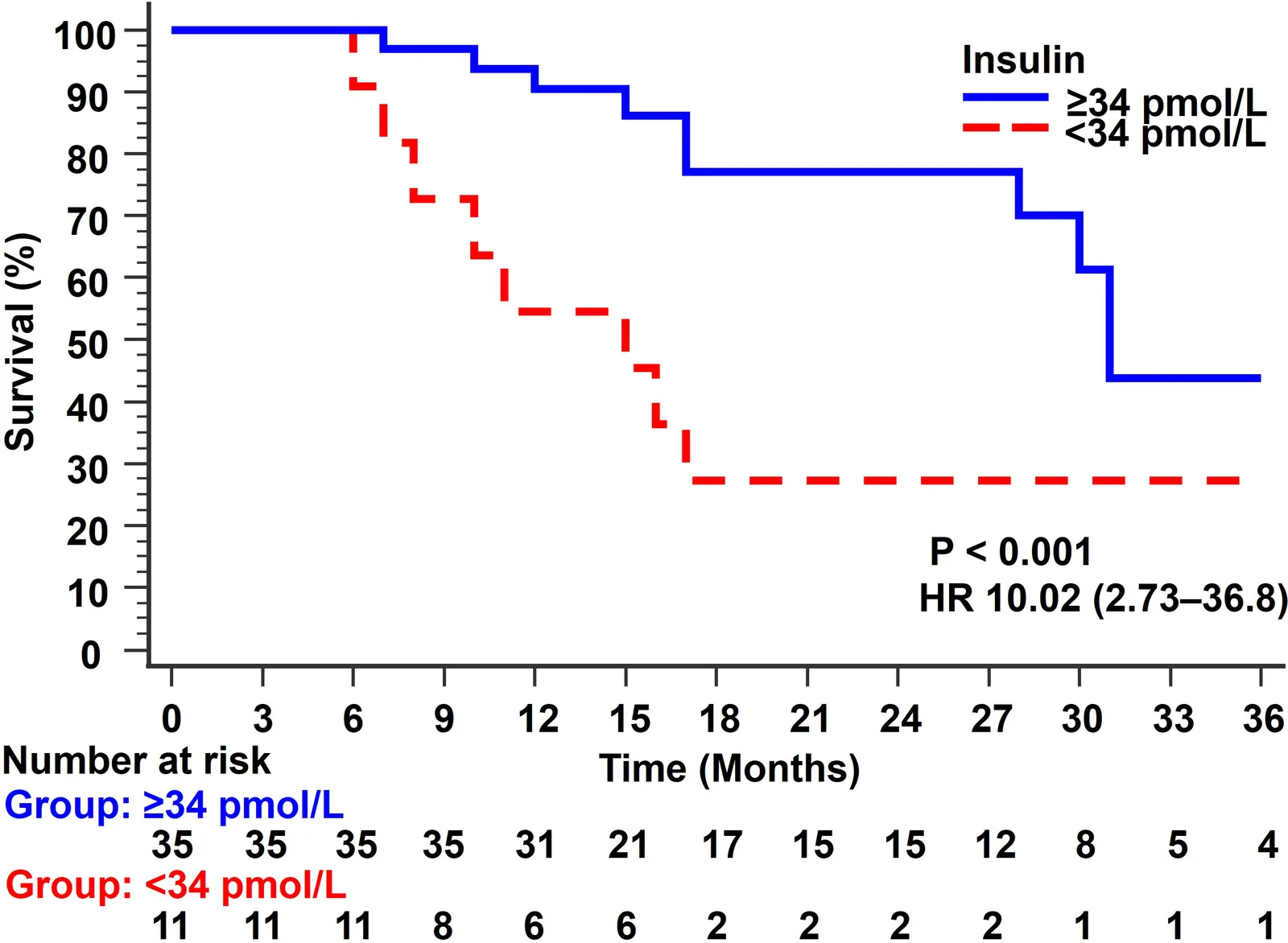
Kaplan–Meier overall survival by fasting insulin level. Patients with insulin > 34 pmol/L (blue line) had significantly better overall survival compared with those with insulin ≤ 34 pmol/L (red line). *P* < 0.001; HR, 10.02; 95% CI, 2.73–36.8. Insulin was known in 46 patients.

Similarly, low fasting insulin (≤34 pmol/L) was strongly associated with poor OS (**Figure 2**). Although insulin data were limited (n = 46), the direction of effect was consistent with multivariable findings. The identified cutoff (34 pmol/L) lies within the lower range of commonly reported fasting insulin reference values.

Ferritin was associated with OS in univariable analysis, while glucose showed only a non-significant trend (**Table 1**).

**Figure 3:**
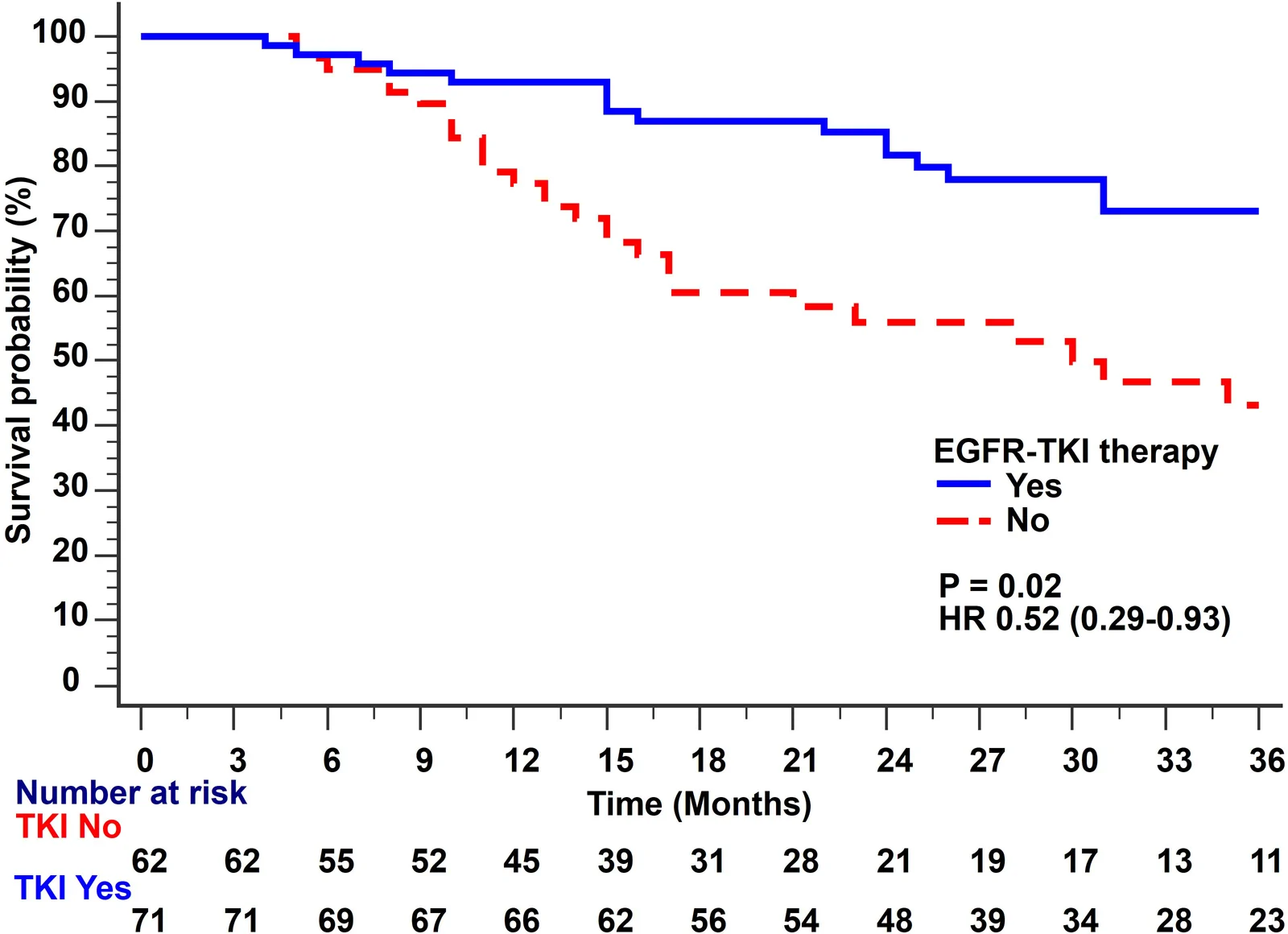
Kaplan–Meier overall survival by EGFR-TKI therapy in all 133 patients. Patients with TKI (blue line) had significantly better overall survival than those without TKI (red dotted line). *P* = 0.02; HR, 0.52; 95% CI, 0.29–0.93.

EGFR–TKI therapy (yes, n = 71 versus no, n = 56) was significantly associated with improved survival (median overall survival 49 versus 30 months, HR = 0.52, 95%CI 0.29-0.93, P = 0.02) (**Figure 3**).

Kaplan–Meier analysis showed that patients receiving EGFR-TKI therapy (n = 71) had the best survival, followed by those receiving non–EGFR-TKI therapy (n = 24), while patients without TKI treatment (n = 32) had the worst survival (72%, 54%, and 36% at 3-year follow-up, respectively). Median survival times were not reached for the EGFR-TKI and non–EGFR-TKI groups, compared with 21 months for patients without TKI treatment.

### Multivariable analysis

In multivariable Cox regression models including BMI, fasting insulin, and treatment variables, EGFR–TKI therapy was associated with improved OS (HR 0.18, P = 0.007), independent of BMI and fasting insulin (**Table 2**).

**Table 2:** Multivariable Cox regression including BMI, fasting insulin, and EGFR-TKI.

Variable	HR	95% CI	P value
BMI <22.1 kg/m² (vs ≥22.1)	3.60	1.22–10.61	0.020
Fasting insulin ≤34 pmol/L (vs >34)	3.72	1.25–11.08	0.018
EGFR–TKI therapy (yes vs no)	0.18	0.05–0.63	0.007

HR, hazard ratio; CI, confidence interval; BMI, body mass index; EGFR, epidermal growth factor receptor; TKI, tyrosine kinase inhibitor.

Patients with low BMI and/or low insulin also derived benefit from EGFR–TKI therapy, but outcomes without EGFR-TKI treatment (n=24 + 32 = 56) had markedly poor survival, with 36-month OS rates for treated versus untreated patients of 88% versus 35%.

In the patients with BMI ≥22.1 kg/m² or (BMI <22.1 kg/m² with insulin >34 pmol/L), EGFR–TKI therapy was also associated with improved survival (HR 0.13, 95% CI 0.04–0.42; P = 0.0006), with corresponding 36-month OS rates of 92% versus 36%.

Given the small number of patients in the low-insulin subgroup (n = 11), these estimates should be interpreted with caution. Other metabolic variables, including glucose, ferritin, and T2DM, were not independently associated with OS after adjustment, and no significant associations were observed for immune checkpoint inhibitor (ICI) or anti-VEGF therapies (**Table 3**).

**Table 3:** Multivariable Cox regression analysis of metabolic and all treatment variables and overall survival.

Variable	HR	95% CI	P value
BMI <22.1 kg/m² (vs ≥22.1)	4.61	1.12–16.93	0.03
Diabetes mellitus (yes vs no)	0.45	0.11–1.83	0.26
Fasting insulin ≤34 pmol/L (vs >34)	3.72	1.25–11.08	0.02
Glucose >5.64 mmol/L (vs ≤5.64)	3.01	0.67–13.53	0.15
Ferritin ≥328 ng/mL (vs <328)	2.40	0.69–8.33	0.17
EGFR–TKI therapy (yes vs no)	0.17	0.04–0.69	0.01
Immunotherapy (yes vs no)	0.58	0.17–1.94	0.38
Anti-VEGF therapy (yes vs no)	1.99	0.69–5.71	0.20

HR, hazard ratio; CI, confidence interval; BMI, body mass index; EGFR, epidermal growth factor receptor; TKI, tyrosine kinase inhibitor.

### Correlation analysis

**Figure 4:**
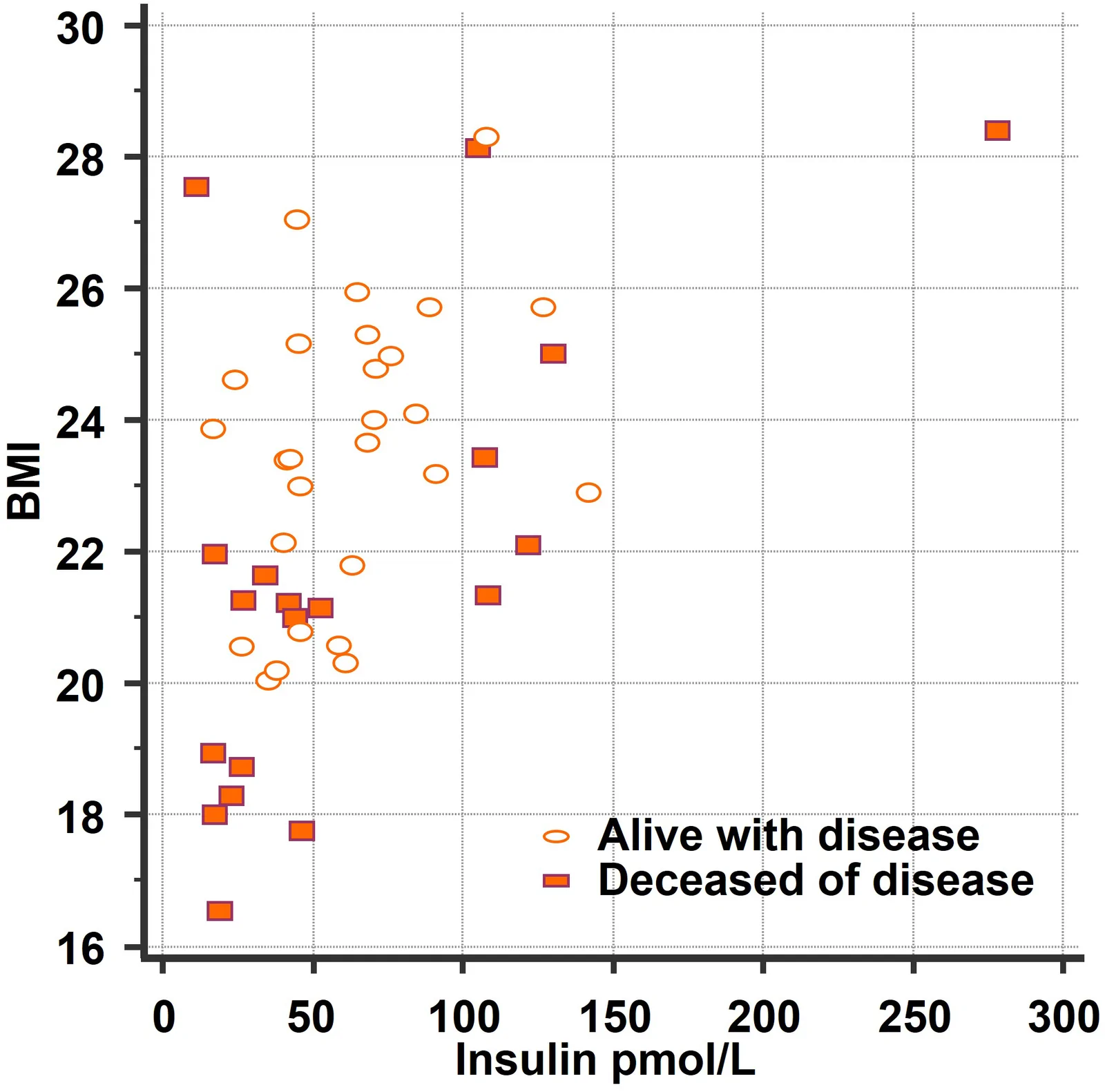
Scatter plot illustrating the relationship between body mass index (BMI), fasting insulin levels and overall survival. Each point represents an individual patient, with colors indicating survival status (alive vs deceased). Lower BMI (<22.1 kg/m²) was generally associated with lower fasting insulin levels. Insulin data were available for n = 46 patients, BMI data for n = 104 patients, and paired BMI–insulin data for 45 patients.

BMI and fasting insulin showed a moderate positive correlation (Spearman r = 0.53, P = 0.0002; n = 45, in 1 case with known insulin, BMI was lacking), indicating that patients with lower BMI tended to have lower insulin levels (**Figure 4**). This association was more pronounced among patients who died compared with those alive at follow-up.

### Propensity score analysis

Propensity score matching yielded 34 well-balanced patient pairs (n = 68), with adequate covariate balance (standardized mean differences <0.1). In the matched cohort, low BMI (<22.1 kg/m²) remained significantly associated with shorter OS, confirming that the observed associations were not explained by baseline imbalances (**Supplementary Tables 6**, **7**).

The survival benefit of EGFR–TKI therapy remained evident after matching.

### Sensitivity analyses

Sensitivity analyses yielded results consistent with the primary analysis. Low BMI and low insulin remained associated with poor overall survival, with similar effect sizes and no change in the direction or statistical significance of associations, demonstrating that the association between low BMI and poor survival was robust.

## Discussion

### Principal findings

In this prospective, homogeneous cohort of patients with stage IV lung adenocarcinoma (LUAD) and ECOG performance status 0–1, low BMI and low fasting insulin identified a metabolically depleted subgroup with particularly poor outcomes, especially among patients not receiving EGFR–TKI therapy. These findings highlight host metabolic status as an important determinant of clinical outcomes beyond established tumor-specific biomarkers.

The ROC-derived BMI cutoff of 22.1 kg/m² lies within the lower range of the normal BMI spectrum and is close to the upper limit defined for Asian populations (22.9 kg/m²) by the World Health Organization. This suggests that even modest reductions in BMI within conventionally normal ranges may have prognostic relevance, extending beyond individuals who are formally underweight. Sensitivity analyses using alternative categorizations (e.g., median and tertiles) yielded consistent findings, supporting the robustness of the observed associations.

Low BMI and low fasting insulin concentrations—markers of host metabolic depletion—were independently associated with shorter overall survival and markedly reduced benefit from EGFR–TKI therapy. Fasting insulin levels in healthy individuals typically fall within a broad reference range (10.4–104.0 pmol/L, depending on assay and population). The threshold identified in this study (≤34 pmol/L) lies at the lower end of this range, supporting the interpretation that very low insulin reflects a state of metabolic depletion rather than normal physiological variation. These findings suggest that systemic energy deficiency, rather than metabolic excess, may be a key determinant of treatment response.

Body mass index (BMI) is closely linked to insulin resistance and systemic metabolic regulation, with higher BMI generally associated with increased insulin resistance and altered insulin/IGF signaling pathways. In lung cancer, this metabolic axis has been increasingly recognized as clinically relevant, with studies demonstrating associations between BMI and outcomes not only in EGFR-mutated disease but also in ALK-rearranged tumors and in patients receiving immune checkpoint inhibitors (21, 25, 39–42). In particular, recent studies have shown that BMI may influence the efficacy of ALK inhibitors and immunotherapy, and that insulin resistance–related metabolic phenotypes are associated with tumor progression and survival in non–small cell lung cancer (39–42). Collectively, these observations support the concept that host metabolic status, including insulin-related pathways, may modulate treatment response across different molecular subtypes of non–small cell lung cancer. Importantly, our findings extend this framework by demonstrating that not only metabolic excess, but also metabolic depletion—reflected by low BMI and low insulin—has important prognostic implications.

### Biological plausibility and mechanistic links

Host metabolic depletion provides a biologically plausible framework for these findings. However, BMI and fasting insulin should be interpreted as indirect proxies of systemic metabolic status rather than direct measures of cachexia or body composition. Insulin and IGF signaling pathways interact with EGFR through downstream PI3K–AKT–mTOR signaling. Reduced insulin levels may therefore influence anabolic signaling pathways relevant to tumor growth and response to EGFR-TKI therapy.

In addition, systemic metabolic depletion is closely linked to sarcopenia and cancer cachexia (43–45), which impair physiologic reserve, reduce substrate availability, and adversely affect treatment tolerance (46). Alterations in mitochondrial function and nutrient metabolism may further compromise the effectiveness of targeted therapies (47–51). Cachexia-associated inflammation, including IL-6/STAT3 signaling, can promote catabolic states and contribute to therapeutic resistance, with elevated ferritin potentially reflecting this inflammatory milieu (47, 48).

Pharmacokinetic factors may also contribute. Systemic inflammation and altered physiologic states can influence drug absorption (49), metabolism, and exposure (47). For example, gastric pH–modifying agents have been shown to significantly affect EGFR–TKI bioavailability (48, 50), underscoring the sensitivity of these agents to host physiological conditions.

Further supporting these links, insulin receptor substrate-1 (IRS-1) has been shown to interact directly with EGFR signaling complexes, providing a molecular interface between insulin/IGF and EGFR pathways (51). Clinical pharmacokinetic studies have demonstrated that systemic inflammation, such as elevated IL-6 levels, may alter osimertinib exposure and correlate with reduced survival (52). In addition, metabolic–oncologic crosstalk involving the IGF-1R/EGFR axis represents a key interface linking metabolic state to therapeutic response in lung cancer (53). Together, these data support the concept that host metabolic depletion may influence overall survival through combined effects on signaling pathways, and inflammation.

### Clinical and methodological implications

These findings suggest that simple, routinely available clinical measures—such as BMI and fasting insulin—may help identify patients at risk of poor outcomes related to metabolic depletion. Incorporating such parameters into clinical assessment could identify patients at risk for poor outcomes despite favorable molecular profiles (i.e., absence of actionable EGFR-mutations) and may support early alternative intervention strategies, including nutritional optimization, metabolic support, and exercise-based rehabilitation.

### EGFR- and other-TKI treatments

During the study period (2020–2024), targeted therapy in China was predominantly based on EGFR-TKIs (primarily gefitinib and osimertinib). Other targeted treatments included KRAS G12C inhibitors, ALK inhibitors (e.g., crizotinib), and more recently RET inhibitors (selpercatinib, pralsetinib), while HER2- and BRAF-mutated tumors were treated with pyrotinib and dabrafenib–trametinib, respectively.

The non–EGFR-TKI group in **Table 1** comprised a biologically heterogeneous population, including patients receiving other targeted therapies as well as those without TKI treatment, which should be considered when interpreting comparisons.

### Strengths, limitations, future directions

Methodological strengths of this study include its prospective design, consecutive patient inclusion, and restriction to a clinically homogeneous cohort with ECOG performance status 0–1. Metabolic variables were systematically assessed with modern equipment, and analyses incorporated predefined multivariable models and propensity score matching, consistent with RECORD-PE reporting standards for observational pharmacoepidemiologic studies (54). Sensitivity analyses addressing missing data yielded results consistent with the primary findings, suggesting limited impact of missing data on overall conclusions.

Several limitations should be acknowledged. Glucose and insulin measurements were not available for all participants. The insulin analysis was based on a limited subset of patients, particularly only 11 with very low insulin values, and should therefore be interpreted as exploratory despite the large observed effect size. Because of the small number of events and the limited size of the low-insulin subgroup, the study had limited power to estimate the insulin effect precisely, and the reported hazard ratios may be unstable. Residual confounding from unmeasured factors, including nutritional intake, body composition, and systemic inflammation, cannot be fully excluded, in spite of the use of propensity score analysis. The observational, single-center design also limits causal inference and generalizability, and these findings must be validated in independent, external cohorts.

Future multicenter studies integrating metabolic, inflammatory, and pharmacokinetic profiling are warranted to clarify the mechanisms underlying these associations. Such studies could determine whether metabolic depletion directly modifies drug exposure, immune response, or both, and may inform personalized treatment strategies in advanced LUAD.

## Conclusions

Low BMI and low fasting insulin identify a metabolically depleted subgroup with poor outcomes in stage IV LUAD, particularly in patients not receiving EGFR–TKI therapy. These findings highlight the importance of host metabolic status in influencing clinical outcomes and suggest that simple, routinely available measures may improve risk stratification. Early nutritional and exercise-based interventions may represent potential strategies to improve outcomes in this vulnerable subgroup.

## Data Availability

The raw data supporting the conclusions of this article will be made available upon reasonable request, from the first corresponding author.

## Supplementary material

Supplementary Figure 1 STROBE flow diagram of patient selection. Consecutive patients diagnosed with stage IV non–small cell lung cancer (NSCLC) at Longhua University Hospital between 2020 and 2024 were screened. After exclusions for ECOG performance status >1, loss to follow-up, missing or inadequate biomarker data, non-adenocarcinoma histology, or survival <3 months, 133 patients with stage IV lung adenocarcinoma (ECOG 0–1) and ≥3 months follow-up were included in the final analysis (follow-up to March 31, 2025).
