# Correction: Body mass index and fasting insulin predict survival and EGFR–TKI benefit in Stage IV lung adenocarcinoma

**DOI:** 10.3389/fonc.2026.1928120

**Published:** 2026-08-10

**Authors:** Huiru Guo, Lingshuang Liu, Jan P. A. Baak

**Affiliations:** 1 Department VI of Medical Oncology, Longhua University Hospital, Shanghai, China; 2 Department of Molecular Digital Pathology, Stavanger University Hospital, Stavanger, Norway

**Keywords:** body mass index, cancer metabolism, EGFR tyrosine kinase inhibitor, fasting insulin (FINS), lung adenocarcinoma, non-small cell lung cancer, overall survival (OS time)

The **Reference** for 5 was erroneously written as: “de Jager VD, Timens W, Bayle A, van den Berg A, van der Wekken AJ, Groen HJM, et al. Future perspective for the application of predictive biomarker testing in advanced- stage non–small-cell lung cancer. Lancet Reg Health Eur. (2024) 38:100839. doi: 10.1016/j.lanepe.2024.100839.” It should be: “de Jager VD, Timens W, Bayle A, Botling J, Brcic L, Büttner R, at al. Future perspective for the application of predictive biomarker testing in advanced stage non-small cell lung cancer. Lancet Reg Health Eur. (2024)38:100839. doi: 10.1016/j.lanepe.2024.100839.”

The **Reference** for 13 was erroneously written as: “Osataphan S, Awidi M, Jan YJ, Gunturu KS, Sundararaman S, Viray R, et al. Association between higher glucose levels and reduced survival in patients with non-small cell lung cancer treated with immune checkpoint inhibitors. Lung Cancer. (2024) 198:108023.” doi: 10.1016/j.lungcan.2024.108023. It should be: “Osataphan S, Awidi M, Jan YJ, Gunturu K, Sundararaman S, Viray H, et al. Association between higher glucose levels and reduced survival in patients with non-small cell lung cancer treated with immune checkpoint inhibitors. Lung Cancer. (2024) 198:108023. doi: 10.1016/j.lungcan.2024.108023.”

The **Reference** for 17 was erroneously written as: “Li L, Yang L, Fan Z, Xue F, Shen Y, Yuan C, et al. Hypoxia-induced GBE1 expression promotes tumor progression through metabolic reprogramming in lung adenocarcinoma. Signal Transduct Target Ther. (2020) 5:54. doi: 10.1038/s41392-020- 0152-8.” It should be: “Li L, Yang L, Fan Z, Xue W, Shen Z, Yuan Y, et al. Hypoxia-induced GBE1 expression promotes tumor progression through metabolic reprogramming in lung adenocarcinoma. Signal Transduct Target Ther. (2020) 5:54. doi: 10.1038/s41392-020-0152-8.”

The **Reference** for 20 was erroneously written as: “Lende TH, Austdal M, Varhaugvik AE, Skaland I, Gudlaugsson E, Kvaløy JT, et al. Preoperative carbohydrate loading in breast cancer patients increases proliferation and is associated with unfavorable outcome. BMC Cancer. (2019) 19:1006. doi: 10.1186/s12885-019-6221-4.” It should be: “Lende TH, Austdal M, Varhaugvik AE, Skaland I, Gudlaugsson E, Kvaløy JT, et al. Influence of pre-operative oral carbohydrate loading vs. standard fasting on tumor proliferation and clinical outcome in breast cancer patients ─ a randomized trial. BMC Cancer. (2019)19:1076. doi: 10.1186/s12885-019-6275-z.”

The **Reference** for 22 was erroneously written as: “Yang SY, Zhao L, Li J, Fang H, Zhang D, Xia L, et al. Type 2 diabetes mellitus is an independent prognostic factor in stage IIIB–IV non-small cell lung cancer patients. Thorac Cancer. (2022) 13:1780–9. doi: 10.1111/1759-7714.14676.” It should be: “Li X, Fang H, Zhang D, Xia L, Wang X, Yang J, et al. Long-term survival analysis of patients with stage IIIB-IV non-small cell lung cancer complicated by type 2 diabetes mellitus: A retrospective propensity score matching analysis. Thorac Cancer. (2022)13:3268-73. doi: 10.1111/1759-7714.14676.”

The **Reference** for 26 was erroneously written as: “Arrieta O, Barrón F, Salinas Padilla MÁ, Avilés-Salas A, Ramıŕez-Tirado LA, Arguelles Jiménez MJ, et al. Effect of metformin plus tyrosine kinase inhibitors compared with tyrosine kinase inhibitors alone in patients with EGFR-mutated lung adenocarcinoma: a phase 2 randomized clinical trial. JAMA Oncol. (2019) 5:e192553. doi: 10.1001/jamaoncol.2019.2553.” It should be: “Arrieta O, Barrón F, Padilla MS, Avilés-Salas A, Ramírez-Tirado LA, Arguelles Jiménez MJ, et al. Effect of metformin plus tyrosine kinase inhibitors compared with tyrosine kinase inhibitors alone in patients with epidermal growth factor receptor-mutated lung adenocarcinoma: a phase 2 randomized clinical trial. JAMA Oncol. (2019)5:e192553. doi: 10.1001/jamaoncol.2019.2553.”

The **Reference** for 34 was erroneously written as: “Burke JF, Hayward RA, Redberg RF. Prospective propensity score matching and inverse probability treatment weighting for observational data in clinical practice. JAMA. (2020) 323:1057–8. doi: 10.1001/jama.2020.0310.” It should be: “Thomas L, Li F, Pencina M. Using Propensity Score Methods to Create Target Populations in Observational Clinical Research. JAMA. (2020) 323:466-7. doi: 10.1001/jama.2019.21558. PMID: 31922529.”

The **Reference** for 45 was erroneously written as: Peixoto da Silva S, Santos JMO, Costa E, Silva C, Gil da Costa RM, Medeiros R. Cancer cachexia and its pathophysiology: links with sarcopenia, anorexia and asthenia. J Cachexia Sarcopenia Muscle. (2020) 11:619–35. doi: 10.1002/jcsm.12528. It should be: “Peixoto da Silva S, Santos JMO, Costa E Silva MP, Gil da Costa RM, Medeiros R. Cancer cachexia and its pathophysiology: links with sarcopenia, anorexia and asthenia. J Cachexia Sarcopenia Muscle. (2020)11:619-35. doi: 10.1002/jcsm.12528.”

The **Reference** for 50 was erroneously written as: “van Leeuwen RWF, Peric R, Hussaarts KGAM, Kienhuis E, IJzerman NS, de Bruijn P, et al. Influence of the acidic beverage cola on the absorption of erlotinib in patients with non-small-cell lung cancer. J Clin Oncol. (2016) 20:1309–14. doi: 10.1200/JCO.2015.65.2560.” It should be: “van Leeuwen RW, Peric R, Hussaarts KG, Kienhuis E, IJzerman NS, de Bruijn P, et al. Influence of the acidic beverage cola on the absorption of erlotinib in patients with non-small-cell lung cancer. J Clin Oncol. (2016)34:1309-14. doi: 10.1200/JCO.2015.65.2560.”

The **Reference** for 51 was erroneously written as: “Takács T, Barna BA, Kiss A, Csaba-Kiss Z, Adamczyk K, Kulka J, et al. Insulin receptor substrate 1 is a novel member of EGFR signalling in pancreatic ductal adenocarcinoma. Gene Expression Patterns. (2024) 45:100186. doi: 10.1016/j.gep.2024.100186.” It should be: “Takács T, László L, Tilajka Á, Novák J, Buday L, Vas V. Insulin receptor substrate 1 is a novel member of EGFR signaling in pancreatic cells. Eur J Cell Biol. (2024)103:151457. doi: 10.1016/j.ejcb.2024.151457.”

The **Reference** for 52 was erroneously written as: “Yokota H, Sato K, Miura M, Nakamura T, Watanabe Y, Ito H, et al. Influence of interleukin-6 on the pharmacokinetics and overall survival after osimertinib therapy in EGFR-mutated non-small cell lung cancer. Cancer Chemother Pharmacol. (2025):in press. doi: 10.1007/s00280-025-04772-x.” It should be: “Yokota H, Sato K, Sakamoto S, Okuda Y, Takeda M, Akamine Y, et al. Influence of interleukin-6 on the pharmacokinetics and pharmacodynamics of osimertinib in patients with non-small cell lung cancer. Cancer Chemother Pharmacol. (2025)95:49. doi: 10.1007/s00280-025-04772-x.”

The **Reference** for 53 was erroneously written as: “Azani A, Smith J, Brown L, Zhao Y, Wong K, Lee H, et al. Targeting the molecular crosstalk between diabetes and lung cancer: the IGF-1R/EGFR axis. Cancer Metab Ther. (2025) 1:45–56. doi: 10.1007/s12672-025-03264-x.” It should be: “Azani A, Avval NA, Meigoli MSS, Imankhan M, Asgari P, Ebrahimisaraj G, et al. Targeting the molecular crosstalk between diabetes and lung cancer for therapeutic intervention. Discov Oncol. (2025)16:1427. doi: 10.1007/s12672-025-03264-x.”

The original version of this article has been updated.

