# Supplementary figures and images for: Body mass index and fasting insulin predict survival and EGFR–TKI benefit in Stage IV lung adenocarcinoma
Source: Front Oncol. 2026 Jun 25;16:1742177. doi: 10.3389/fonc.2026.1742177 (PMC13347122; doi:10.3389/fonc.2026.1742177)

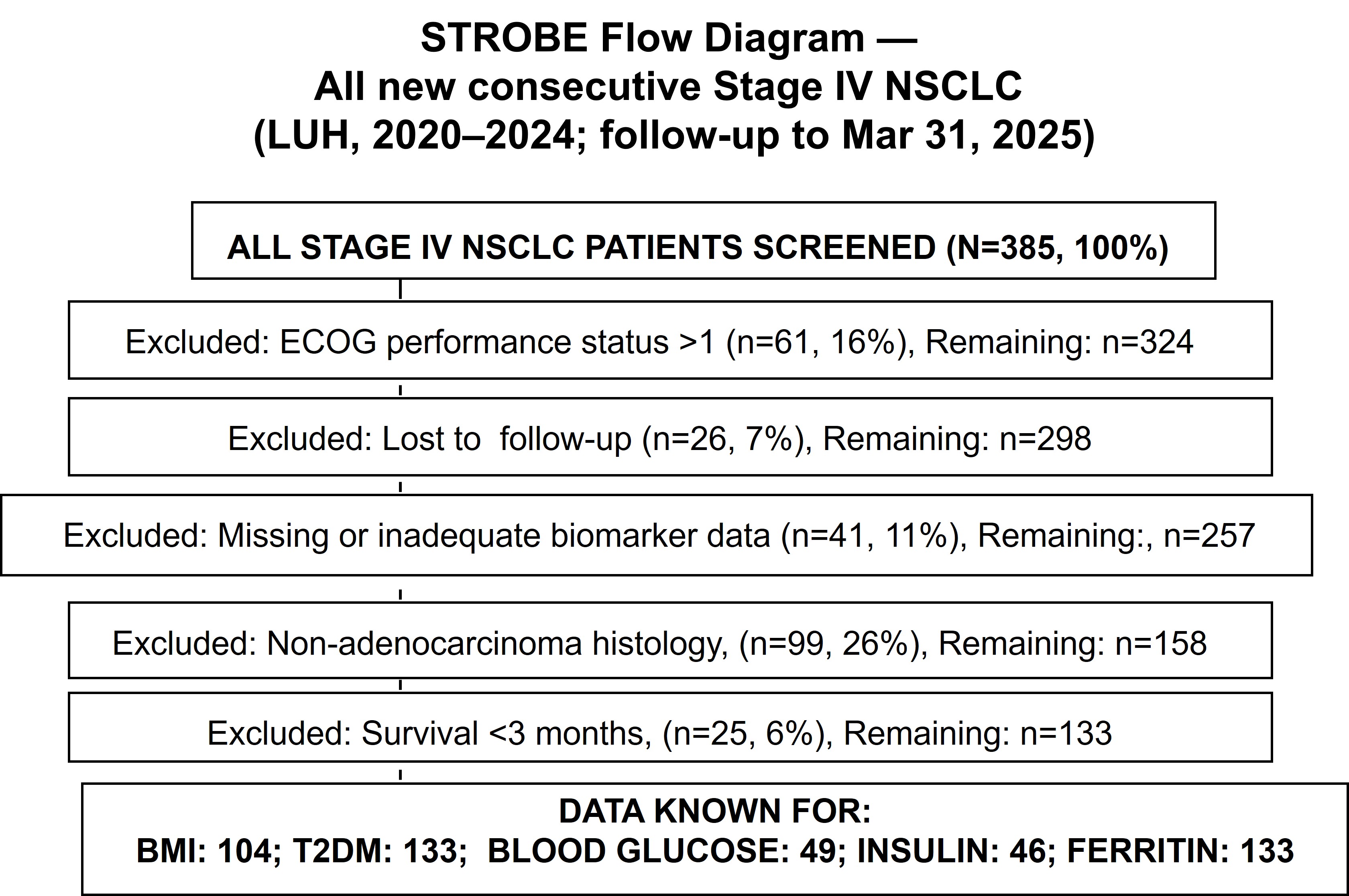

Supplement: Supplementary Figure 1 — STROBE flow diagram of patient selection. Consecutive patients diagnosed with stage IV non–small cell lung cancer (NSCLC) at Longhua University Hospital between 2020 and 2024 were screened. After exclusions for ECOG performance status >1, loss to follow-up, missing or inadequate biomarker data, non-adenocarcinoma histology, or survival <3 months, 133 patients with stage IV lung adenocarcinoma (ECOG 0–1) and ≥3 months follow-up were included in the final analysis (follow-up to March 31, 2025). [file Image1.jpeg]
