# Supplementary material for: Body mass index and fasting insulin predict survival and EGFR–TKI benefit in Stage IV lung adenocarcinoma
Source: Front Oncol. 2026 Jun 25;16:1742177. doi: 10.3389/fonc.2026.1742177 (PMC13347122; doi:10.3389/fonc.2026.1742177)
Supplement: Supplementary file 2 [file Table1.docx]

# **Supplementary FILE**

The study design adhered to STROBE guidelines (Supplementary Figure S1). The primary endpoint was overall survival (OS), defined from diagnosis to death. Survival was assessed with Kaplan–Meier estimates and Cox models, with proportional hazards assumptions tested. Key covariates were age, sex, TNM stage (IVA vs IVB), EGFR mutation status, body mass index (BMI), type 2 diabetes, and ferritin.

# Data supplementary Table S1 shows the standardized mean difference (SMD) calculation methods by covariate type and data supplementary. Table S2 shows the propensity score check list.

**Supplementary Tables**

**Supplementary Table S1.** Targeted Therapies and Corresponding Genomic Alterations

| Therapy | Target | Events / Total | Median OS (months) |
| --- | --- | --- | --- |
|  |  |  |  |
| EGFR-TKIs (gefitinib, osimertinib) | EGFR | 23 / 71 | 49.0 |
| KRAS G12C inhibitors | KRAS G12C | 4 / 10 | 27.0 |
| Crizotinib | ALK (± ROS1) | 2 / 8 | 60.0 |
| Pyrotinib | HER2 (ERBB2) | 1 / 2 | 30.0 |
| Dabrafenib + Trametinib | BRAF V600E | 0 / 2 | 18.0 |
| Selpercatinib / Pralsetinib | RET | 1 / 2 | 23.0 |

**Supplementary table S2.** Details Equipment, test methods and normal ranges for glucose, ferritin and Insulin

| **Biomarker** | **Equipment / Test Method** | **Company** | **Reference**  **Range** |
| --- | --- | --- | --- |
| Glucose | Beckman AU5800 | Beckman | 3.9-6.1 mmol/L |
| Ferritin | Cobas 8000 analyzer | Roche | Men: 30 – 400 µg/L  Women: 13 – 150 µg/L |
|  |  |  |  |
| Insulin | Cobas 8000 (Elecsys assay) | Roche | 18 – 173 pmol/L |

**Supplementary Table S3.** Standardized mean difference (SMD) calculation methods by covariate type.

| **Type of covariate** | **Formula** | **Description** | **Explanation** |
| --- | --- | --- | --- |
| **Continuous covariates** | SMD = (Mean₁ - Mean₀) / √[(Var₁ + Var₀) / 2] | Mean₁, Mean₀: group means; Var₁, Var₀: variances | Difference in group means divided by pooled SD (Cohen’s d). |
| **Categorical covariates (2 categories)** | SMD = (P₁ - P₀) / √[P(1-P)], *P* = pooled proportion | P₁, P₀: proportions in groups | Difference in proportions divided by SD of pooled proportion. |
| **Categorical covariates (>2 categories)** | SMD_overall = √[Σ(P₁ᵢ - P₀ᵢ)² / K] | P₁ᵢ, P₀ᵢ: category proportions; K = number of categories | Root mean squared difference in proportions across categories. |

Note: The standardized mean difference (SMD) measures group differences in standard deviation units and is widely used to assess covariate balance in observational studies, particularly with propensity score methods. Threshold for good balance: |SMD| < 0.10. Unlike P values, the SMD is independent of sample size.

**Supplementary Table S4.** Quality checklist of propensity score analysis.

| **Item** | **Recommendation** | **Relevant Details** | **Reported in Section** |
| --- | --- | --- | --- |
| **Preparation for PS Analysis** |  |  |  |
| 1. | Point out scientific background | BMI as prognostic/predictive biomarker in stage IV LUAD | Abstract, Introduction |
| 2. | Indicate key study design components | Prospective observational cohort; PS matching to reduce confounding | Methods |
| 3. | State study objectives | Evaluate BMI as independent predictor of survival and treatment modifier | Introduction |
| 4. | Describe data sources and variables | Consecutive patients, standardized data collection, biomarker measurements | Methods |
| **PS Model Building** |  |  |  |
| 5. | Select variables for PS model | Age, sex, TNM stage, EGFR mutation, ferritin (baseline factors only) | Methods |
| 6. | Decide PS estimation method | Logistic regression for propensity estimation | Methods |
| 7. | Evaluate overlap | Adequate common support confirmed across groups | Data Supplement |
| 8. | Present initial balance diagnostics | SMD reported before and after matching | Data supplement |
| **Application of PS Methods** |  |  |  |
| 9. | Specify analysis type | Association estimates (not causal treatment effects) for prognostic biomarker | Not applicable |
| 10. | State PS method used | 1:1 nearest-neighbor matching, caliper 0.1 | Methods |
| 10a) | Detail matching strategy | 1:1 matching ratio with strict caliper | Methods |
| 10b) | Management of extreme weights | Not applicable (matching used, not weighting) | - |
| 10c) | Provide matching details | 34 patients retained from 101 after matching (SMD <0.1, adequate overlap) | Methods, Suppl. Tables |
| 10d) | Describe stratification | Overlap assessment only, not for matching | Data Supplement |
| 11. | Present balance diagnostics post-matching | SMD <0.10 achieved for all covariates | Results, Suppl. Tables |
| 12. | State outcome model | Cox regression, Kaplan-Meier survival analysis | Methods, Results |
| 14. | Report effects for all datasets | Full and matched cohort results presented | Results, Tables 1-2, Figures |

**Supplementary Table S5.** Classification accuracy of BMI <22.1 vs ≥ 22.1 of the 104 patients with known BMI in the whole patients´ cohort (n=104). BMI <22.1 is a positive test for high risk of death (“Deceased of disease” = condition present). Sensitivity: 56.8% (21 / 37), Specificity: 62.7% (42 / 67), Positive Predictive Value (PPV): 45.7% (21 / 46), Negative Predictive Value (NPV): 72.4% (42 / 58), Overall Correct (Accuracy): 60.5% (63 / 104).

| **Outcome** | **≥22.1** | **<22.1** | **Total (%)** |
| --- | --- | --- | --- |
| **Alive at last follow-up** | 42 | 25 | 67 (64%) |
| **Deceased of disease** | 16 | 21 | 37 (36%) |
| **Total (%)** | 58 (56%) | 46 (44%) | 104 |

**Supplementary Table S6.** Baseline demographic variables of the study population. Analysis limited to 104 patients with BMI data (29 excluded due to missing data). SMD < 0.10 indicates acceptable balance. n=59/46 pre-PSM and 32/32 post-PSM. Probability of no difference is 2-sided.

Before PSM

| **Variable** | **BMI ≥22.1** | **N1** | **P1** | **BMI**  **< 22.1** | **N2** | **P2** | **SMD)** | **Probability of no difference** |
| --- | --- | --- | --- | --- | --- | --- | --- | --- |
| **Age (years)** | 64 ± 9 | 55 |  | 67 ± 10 | 46 |  | -0.317 | 0.11 |
| **Sex (M/F)** | 34 (62%) | 55 | 0.62 | 35/11 | 46 | 0.76 | -0.312 | 0.12 |
| **TNM stage** | IV-A:24, IV-B:31 | 55 | 0.44 | IV-A:28, IV-B:18 | 46 | 0.61 | -0.350 | 0.08 |
| **EGFR mutations** | 33 (60%) | 55 | 0.60 | 26 (61%) | 46 | 0.57 | 0.071 | 0.72 |
| **Ferritin ≥328** | 21 (38%) | 55 | 0.38 | 21 (46%) | 46 | 0.46 | -0.152 | 0.44 |

After PSM

| **Variable** | **≥22.1 (n= 34)** | **N1** | **P1** | **< 22.1 (n=34)** | **N2** | **P2** | **SMD (Cohen´s D)** | **Probability of no difference** |
| --- | --- | --- | --- | --- | --- | --- | --- | --- |
| **Age (years)** | 66 ± 7 | 34 |  | 65 ± 7 | 34 |  | 0.143 | 0.55 |
| **Sex (M/F)** | 22/12 | 34 | 0.65 | 19/15 | 34 | 0.56 | 0.181 | 0.45 |
| **TNM stage** | IV-A:15, IV-B:19 | 34 | 0.44 | IV-A:15, IV-B:19 | 34 | 0.44 | 0.000 | 1.00 |
| **EGFR mutations** | 12 (67%) | 34 | 0.67 | 11 (59%) | 34 | 0.59 | 0.184 | 0.45 |
| **Ferritin ≥328** | 13 (38%) | 34 | 0.38 | 15 (44%) | 34 | 0.44 | -0.120 | 0.62 |

**Supplementary Table S7.** Kaplan–Meier survival analysis comparing full and matched datasets ( BMI, ≥22.1, <22.1).

| **Outcome** | **Full dataset (n=104)** | **PSM dataset (n=68)** |
| --- | --- | --- |
| **Median survival (months)** | Not reached (<22.1), 24.1 (≥22.1) | Not reached (≥ 22.1),  31.0 (<22.1) |
| **Mean survival (months)** | 50.1 vs 26.9 | 44.6 vs 35.0 |
| **Restricted Mean Survival Time (36 mo)** | 46.0 vs 26.7 | 46.9 vs 29.8 |
| **Hazard ratio (95% CI)** | 2.51 (1.12–5.60) | 2.51 (1.12-5.60) |
| **Log-rank *P*** | < 0.001 | 0.03 |

Abbreviation: 95% CI, 95% confidence interval. No ferritin component mentioned.

# **Supplementary Figure legend**

**Supplementary Figure S1. STROBE flow diagram of patient selection.**
Consecutive patients diagnosed with stage IV non–small cell lung cancer (NSCLC) at Longhua University Hospital between 2020 and 2024 were screened. After exclusions for ECOG performance status >1, loss to follow-up, missing or inadequate biomarker data, non-adenocarcinoma histology, or survival <3 months, 133 patients with stage IV lung adenocarcinoma (ECOG 0–1) and ≥3 months follow-up were included in the final analysis (follow-up to March 31, 2025).

# **Abbreviations**

BMI, body mass index; CI, confidence interval; EGFR, epidermal growth factor receptor; ECOG, Eastern Cooperative Oncology Group; HR, hazard ratio; OS, overall survival; PSM, propensity score matching; SMD, standardized mean difference; PPV, positive predictive value; NPV, negative predictive value.
